# Distinct Superficial Epidermal Expression Patterns of IL-19 and IL-37 in Atopic Dermatitis

**DOI:** 10.3390/ijms27198741

**Published:** 2026-09-30

**Authors:** Anna Zaryczańska, Krzysztof Sitko, Stefan Tukaj, Magdalena Trzeciak

**Affiliations:** 1 Department of Dermatology, Venereology and Allergology, Medical University of Gdansk, 80-214 Gdansk, Poland; 2 Department of Plant Experimental Biology and Biotechnology, Faculty of Biology, University of Gdansk, 80-308 Gdansk, Poland; 3 Laboratory of Cellular and Molecular Immunology, Department of Molecular Biology, Faculty of Biology, University of Gdansk, 80-308 Gdansk, Poland

**Keywords:** atopic dermatitis, IL19, IL37, tape stripping, RT-qPCR, epidermis, non-lesional skin, cytokine regulation

## Abstract

Atopic dermatitis (AD) is characterized by heterogeneous immune activation extending beyond clinically apparent lesions. Interleukin-19 (IL-19) has been implicated in cytokine-responsive keratinocyte activation, whereas interleukin-37 (IL-37) has endogenous anti-inflammatory functions. Their superficial epidermal expression patterns in lesional and clinically non-lesional AD skin have not been extensively characterized within the same cohort. To compare *IL19* and *IL37* mRNA expression in lesional and clinically non-lesional, lesion-adjacent AD skin with healthy control skin and to explore associations between transcript abundance and clinical characteristics. In this exploratory cross-sectional study, tape strip samples were collected from lesional and clinically non-lesional skin of 32 patients with AD and from corresponding skin of 35 healthy controls.  *IL19* expression did not differ significantly among healthy control, lesional, and clinically non-lesional AD skin in the overall cohort. In contrast, *IL37* transcript abundance was significantly higher in lesional AD skin than in healthy control skin (*p* = 0.0002) and was also increased in clinically non-lesional skin (*p* = 0.0387). In severity-stratified analyses, lesional *IL37* expression was increased in both moderate and severe-to-very severe AD, whereas lower lesional *IL19* expression relative to healthy control skin was observed in severe-to-very severe AD (*p* = 0.0412). Lesional *IL19* expression showed an inverse association with EASI severity category (*p* = 0.045), while lesional *IL37* expression was positively associated with sleep-disturbance scores (*p* = 0.0203). The two transcripts showed distinct expression patterns in this cohort. Increased *IL37* mRNA was compatible with, but did not establish, a compensatory response extending into lesion-adjacent clinically non-lesional skin. Overall *IL19* upregulation was not detected, and lower lesional expression was observed in severe-to-very severe AD. However, these exploratory observations do not establish cytokine function or validate either transcript as a stand-alone biomarker.

## 1. Introduction

Atopic dermatitis (AD) is a chronic, relapsing, intensely pruritic inflammatory skin disease affecting children and adults. Its pathogenesis results from reciprocal interactions among epidermal barrier dysfunction, immune dysregulation, genetic susceptibility, environmental exposures, and cutaneous microbial alterations. Although type 2 inflammation, particularly IL-4- and IL-13-mediated signaling, constitutes the central immunological axis of AD, the disease is molecularly heterogeneous, with variable contributions from Th17-, Th22-, and Th1-associated pathways. Importantly, AD-related abnormalities are not confined to visible eczematous lesions. Clinically non-lesional skin may display subclinical immune activation, impaired terminal keratinocyte differentiation, and altered expression of barrier-related genes, indicating that the molecular disease field extends beyond clinically apparent inflammation [1,2,3]. These observations support compartment-resolved molecular assessment of both lesional and clinically unaffected AD skin.

Within this complex epidermal–immune network, interleukin-19 (IL-19) has emerged as a candidate mediator and molecular readout of inflammation-associated keratinocyte activation. IL-19 belongs to the IL-20 cytokine subfamily and is produced by activated immune cells, particularly monocytes, as well as by epithelial cells, including keratinocytes. It signals through the heterodimeric IL-20Rα/IL-20Rβ receptor complex, supporting local communication between immune and epithelial compartments. Keratinocytes can both produce IL-19 and respond to IL-20-subfamily signaling, suggesting autocrine and paracrine functions within the epidermis [4,5]. Cytokines central to AD directly regulate this pathway: IL-4 induces keratinocyte *IL19* transcription through STAT6-dependent signaling, while IL-17A also potently increases epidermal *IL19* expression [6,7]. Increased *IL19* mRNA and IL-19 protein abundance have been reported in lesional AD skin, and elevated circulating IL-19 concentrations have been associated with clinical disease activity [7,8]. Together, these findings implicate IL-19 in cytokine-responsive epidermal inflammation, although local transcript abundance, skin protein expression, and circulating cytokine concentrations represent distinct biological readouts.

IL-37 represents a biologically distinct component of cutaneous immune regulation. Unlike most members of the IL-1 family, IL-37 predominantly suppresses excessive innate and adaptive immune activation. Its regulatory effects involve complementary intracellular and extracellular mechanisms. Intracellular IL-37 can associate with SMAD3 and modify inflammatory gene transcription, whereas extracellular IL-37 binds IL-18Rα and recruits the inhibitory co-receptor IL-1R8, also known as SIGIRR, initiating a broad anti-inflammatory signaling program [9,10]. In human skin, IL-37 has been localized predominantly to differentiated keratinocytes in the upper epidermis and linked to epidermal differentiation and barrier-associated proteins. Biopsy-based transcriptomic and immunohistochemical studies have demonstrated reduced *IL37* expression and IL-37 protein abundance in AD skin [11]. Clinical findings have nevertheless been inconsistent: both increased and reduced circulating IL-37 concentrations have been reported in patients with AD [12,13]. This variability may reflect differences in disease phenotype, treatment exposure, assay methodology, and the biological compartment examined. It may also indicate that compensatory transcription, protein abundance, receptor availability, and functional anti-inflammatory signaling do not necessarily change in parallel.

IL-19 and IL-37 belong to different cytokine families, engage unrelated receptor systems, and should not be considered direct molecular antagonists. Their parallel assessment may nevertheless provide complementary information about cytokine-responsive epidermal inflammation and endogenous regulation. In an earlier tape strip study of children younger than five years with AD of less than six months’ duration, *IL19* was increased, whereas *IL37* and other negative immune regulators were reduced relative to healthy skin; abnormalities were found in both lesional and clinically non-lesional compartments [14]. It remains uncertain whether these patterns are also observed in older children and adults with different clinical and treatment histories. Measuring both transcripts in the same participants and skin compartments permits a focused comparison within a shared sampling framework without implying a direct interaction between the cytokines.

Tape stripping provides a minimally invasive and repeatable approach to collecting stratum corneum-derived material for superficial epidermal molecular profiling. Sequential application and removal of adhesive discs permits analysis of recoverable transcripts without obtaining a full-thickness skin biopsy. Studies in AD have detected immune, itch-related, and barrier-associated abnormalities in lesional and clinically non-lesional skin [14,15]. Applications extend beyond AD: He et al. identified distinct immune and barrier profiles in AD and psoriasis [15], while Sølberg et al. characterized the transcriptome of hand eczema and identified differences between subgroups, including allergic and irritant contact dermatitis [16]. These findings support biomarker discovery and the development of molecular classification approaches, rather than establish a universally applicable diagnostic test. Tape strip-derived RNA and biopsy-derived RNA represent overlapping but different tissue compartments, as demonstrated by direct paired comparisons [17]. The present study therefore evaluates superficial epidermal expression patterns and clinical associations, not a diagnostic classifier.

Given the heterogeneity of previous findings across age groups, biological compartments, and analytical approaches, the study hypotheses are stated in non-directional terms, consistent with the exploratory design. We hypothesized that relative *IL19* and *IL37* transcript abundance would differ among superficial epidermal samples from lesional AD skin, lesion-adjacent clinically non-lesional AD skin, and healthy control skin in a cohort of older children and adults. We further hypothesized that within-patient lesional and clinically non-lesional transcript abundance might differ and that transcript levels might be associated with disease severity at sampling, pruritus and sleep disturbance, transepidermal water loss, and the demographic and atopy-related characteristics specified in the study outcomes. Age- and severity-stratified analyses were exploratory. The study assessed expression patterns and clinical associations rather than cytokine function or mechanistic interaction.

## 2. Results

Relative transcript abundance was expressed as log2-transformed fold change (log2FC) relative to the healthy-control reference, as described in the Materials and Methods. The primary cross-sectional analysis included paired lesional and clinically non-lesional samples from 32 patients with AD and corresponding skin samples from 35 healthy controls.

### 2.1. Overall Superficial Epidermal Expression of IL19 and IL37

**Figure 1:**
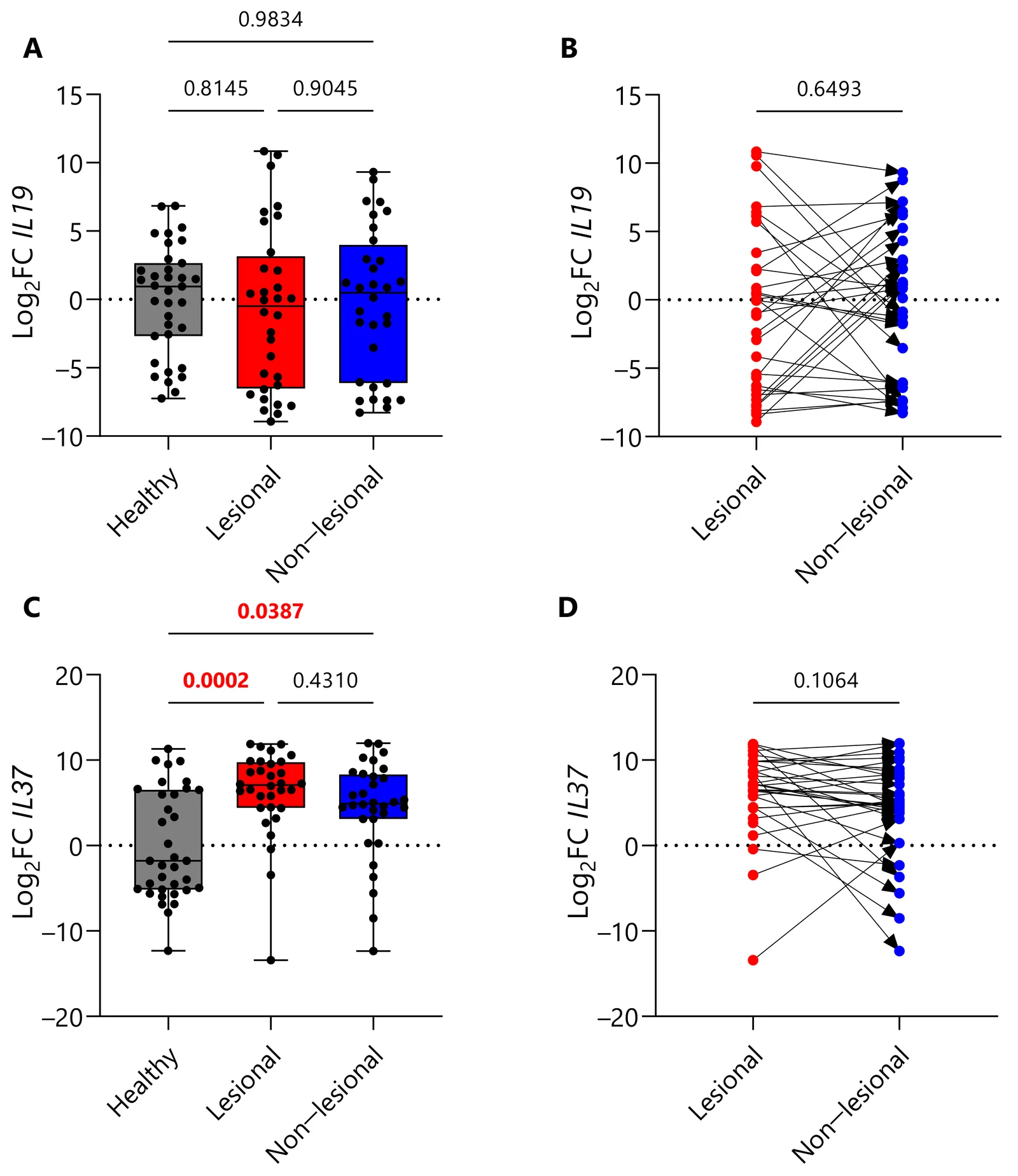
Overall superficial epidermal expression of *IL19* and *IL37*. Relative transcript abundance is expressed as log2-transformed fold change (log2FC) relative to the healthy-control reference. Healthy controls, n = 35; patients with AD, n = 32, with paired lesional and clinically non-lesional samples. (**A**) *IL19*: healthy control vs. lesional skin (*p* = 0.8145), healthy control vs. clinically non-lesional skin (*p* = 0.9834), and lesional vs. clinically non-lesional skin (*p* = 0.9045) were non-significant. (**B**) The paired lesional–non-lesional *IL19* comparison was non-significant (*p* = 0.6493). (**C**) *IL37* was higher in lesional (*p* = 0.0002) and clinically non-lesional (*p* = 0.0387) AD skin than in healthy control skin; the lesional–non-lesional group comparison was non-significant (*p* = 0.4310). (**D**) The paired lesional–non-lesional *IL37* comparison was non-significant (*p* = 0.1064). *p* < 0.05 was considered significant and is represented in red.

Relative IL19 transcript abundance did not differ significantly among healthy control skin, lesional AD skin, and clinically non-lesional AD skin (*p* > 0.05; Figure 1A). The paired within-patient analysis likewise showed no significant difference between lesional and clinically non-lesional skin (*p* > 0.05; Figure 1B), and individual paired values varied in both directions. In contrast, relative IL37 transcript abundance was higher in lesional AD skin than in healthy control skin (*p* = 0.0002) and was also higher in clinically non-lesional AD skin than in healthy control skin (*p* = 0.0387; Figure 1C). No significant difference was detected between lesional and clinically non-lesional AD skin in either the multiple-group comparison or the paired within-patient analysis (*p* > 0.05; Figure 1C,D).

### 2.2. IL19 and IL37 Expression According to AD Severity

**Figure 2:**
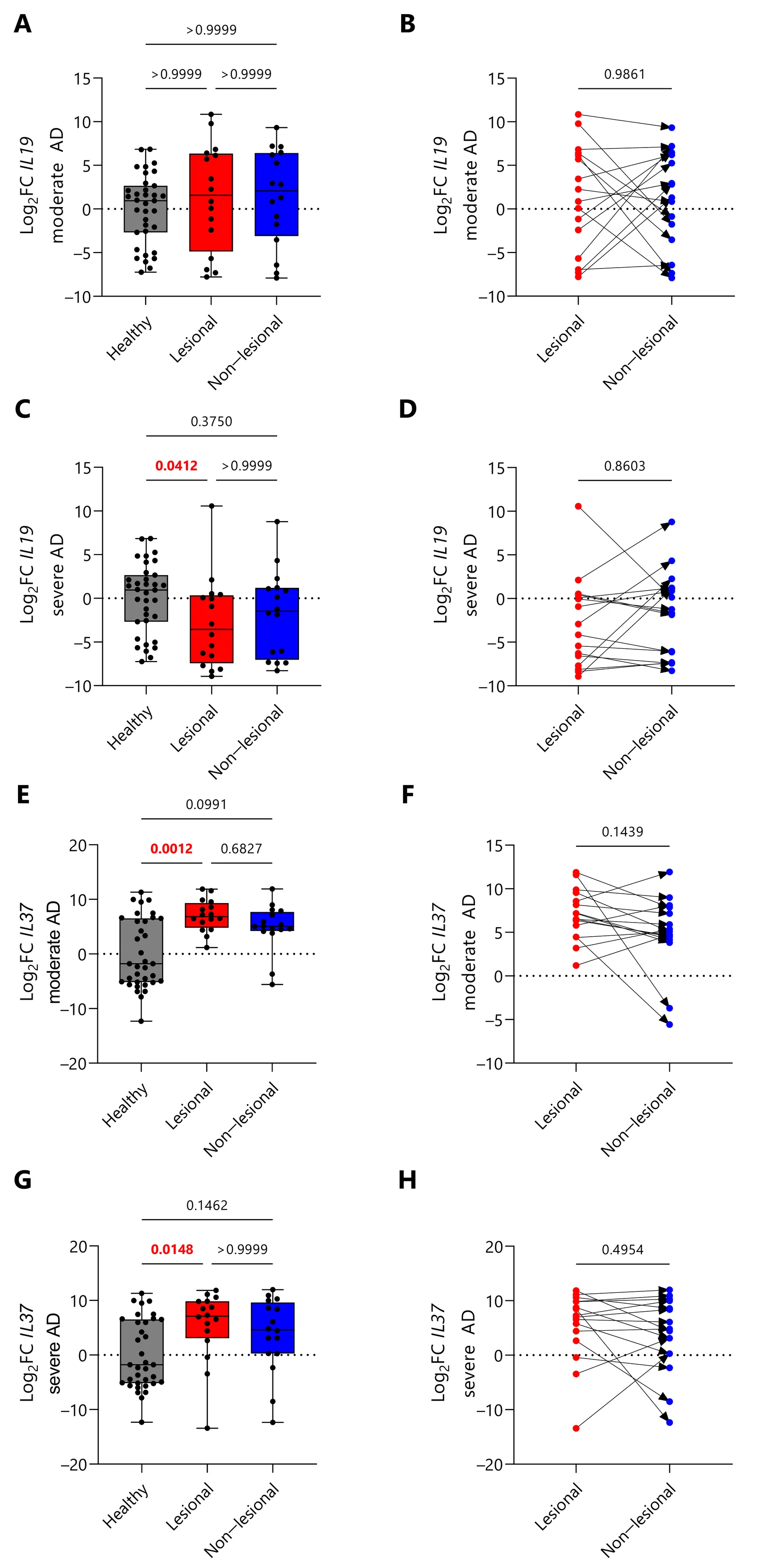
Expression patterns of *IL19* and *IL37* according to AD severity. Relative transcript abundance is shown as log2FC versus the healthy-control reference. (**A**) Moderate AD, *IL19*: no significant effects were reported. (**B**) The paired lesional–non-lesional *IL19* comparison in moderate AD was non-significant. (**C**) Severe-to-very severe AD, *IL19*: was lower in lesional skin compared to healthy control (*p* = 0.0412). (**D**) The paired lesional–non-lesional *IL19* comparison was non-significant. (**E**) Moderate AD, *IL37*: was higher in lesional skin compared to healthy control (*p* = 0.0012). (**F**) The paired lesional–non-lesional *IL37* comparison was non-significant. (**G**) Severe-to-very severe AD, *IL37*: was higher in lesional skin compared to healthy control (*p* = 0.0148). (**H**) The paired lesional–non-lesional *IL37* comparison was non-significant. *p* < 0.05 was considered significant and is represented in red.

The severity-stratified analysis included 16 patients with moderate AD and 16 patients with severe-to-very severe AD. In the moderate AD subgroup, relative *IL19* transcript abundance did not differ significantly among healthy control, lesional, and clinically non-lesional skin (*p* > 0.05; Figure 2A), and the paired within-patient comparison was also non-significant (*p* > 0.05; Figure 2B). In the severe-to-very severe subgroup, lesional *IL19* transcript abundance was lower than in healthy control skin (*p* = 0.0412; Figure 2C). Clinically non-lesional *IL19* transcript abundance did not differ from healthy control skin, and no significant difference between the two AD compartments was identified in either the multiple-group or paired analysis (*p* > 0.05; Figure 2C,D).

Lesional IL37 transcript abundance was higher than in healthy control skin in both the moderate (*p* = 0.0012; Figure 2E) and severe-to-very severe (*p* = 0.0148; Figure 2G) subgroups. In both severity strata, clinically non-lesional IL37 transcript abundance did not differ significantly from healthy control skin, and no significant difference was observed between lesional and clinically non-lesional skin in either the multiple-group or paired within-patient analysis (*p* > 0.05; Figure 2E–H).

### 2.3. Age-Stratified Expression Patterns

**Figure 3:**
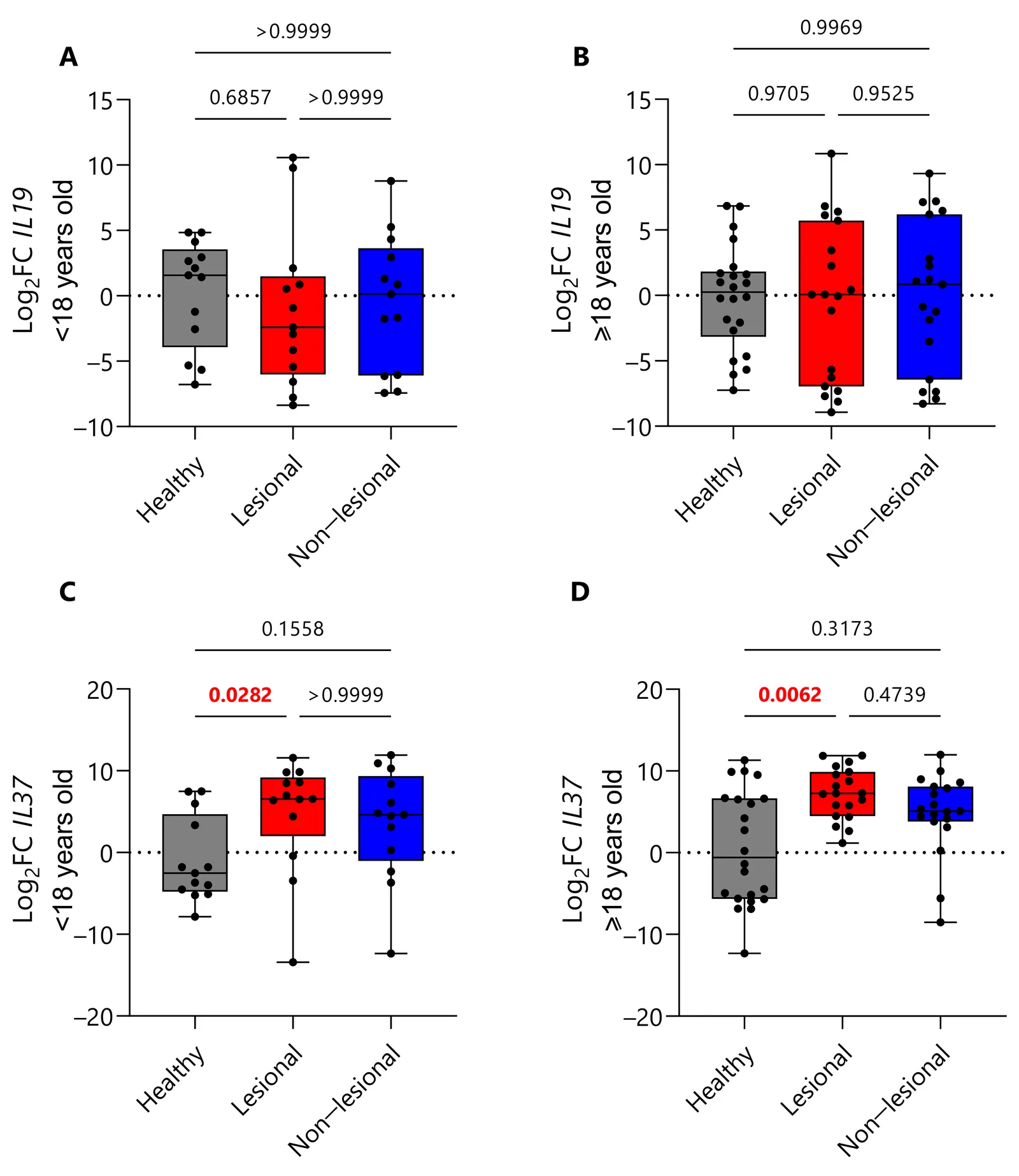
Age-stratified expression patterns of *IL19* and *IL37*. Relative transcript abundance is expressed as log2FC relative to the healthy-control reference. (**A**) Participants younger than 18 years, *IL19*: no significant effects were reported. (**B**) Participants aged 18 years or older, *IL19*: no significant effects were reported. (**C**) Participants younger than 18 years, *IL37*: was higher in lesional skin compared to healthy control (*p* = 0.0282) (**D**) Participants aged 18 years or older, *IL37*: was higher in lesional skin compared to healthy control (*p* = 0.0062). *p* < 0.05 was considered significant and is represented in red.

The age-stratified analysis included 13 patients with AD and 13 healthy controls younger than 18 years, and 19 patients with AD and 22 healthy controls aged 18 years or older. Relative *IL19* transcript abundance did not differ significantly among healthy control, lesional, and clinically non-lesional skin in either age stratum (*p* > 0.05; Figure 3A,B).

Lesional *IL37* transcript abundance was higher than in the corresponding age-stratified healthy control group among participants younger than 18 years (*p* = 0.0282; Figure 3C) and among those aged 18 years or older (*p* = 0.0062; Figure 3D). In both age strata, clinically non-lesional *IL37* transcript abundance did not differ significantly from healthy control skin, and no significant difference was observed between lesional and clinically non-lesional AD skin (*p* > 0.05; Figure 3C,D).

### 2.4. Exploratory Correlation Analyses

**Figure 4:**
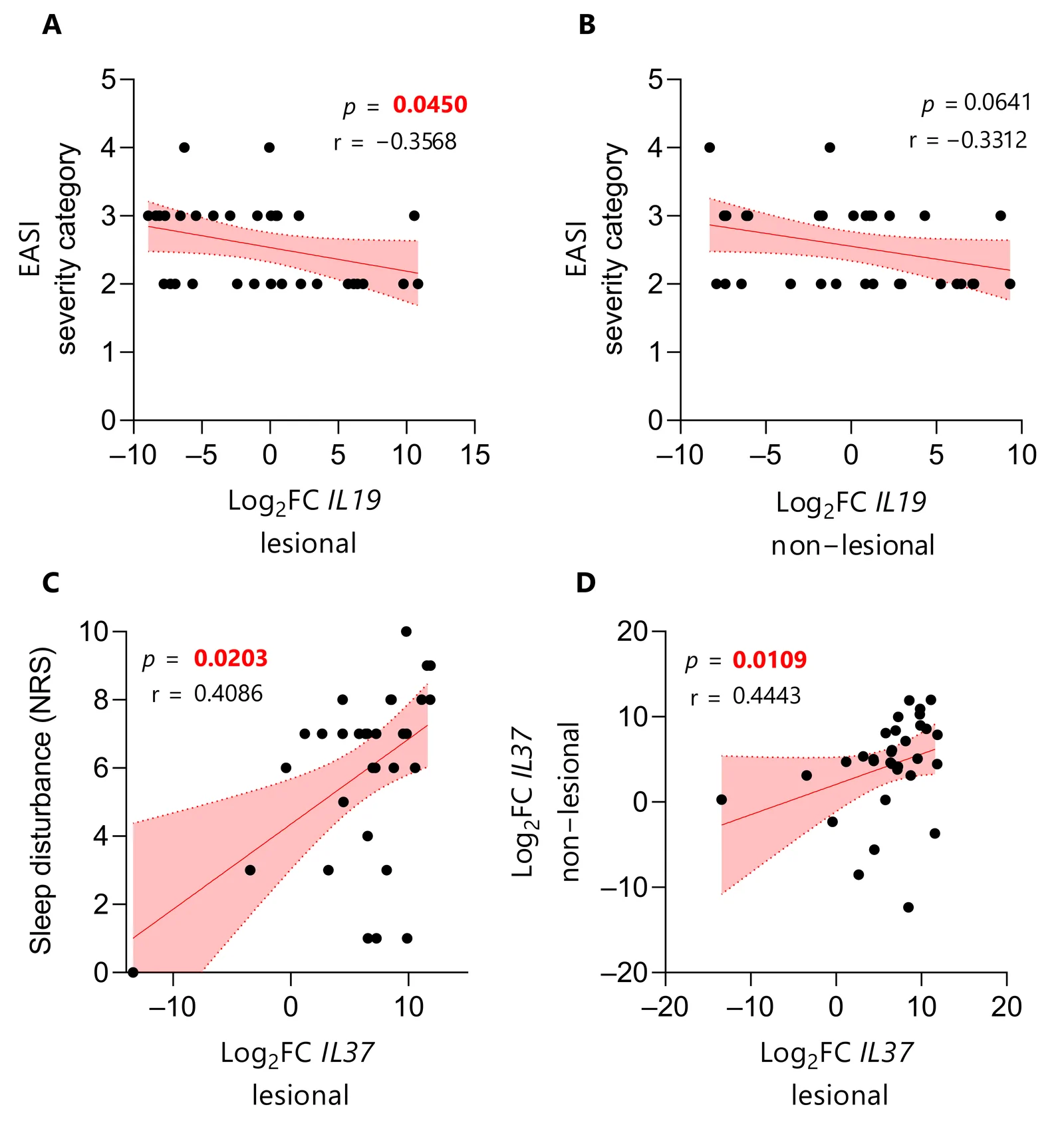
Exploratory associations involving *IL19* and *IL37*. Spearman analyses used log2FC values in the full AD cohort (n = 32); *p*-values are nominal. (**A**) Lesional *IL19* versus EASI severity category (r = −0.3568; *p* = 0.0450). (**B**) Clinically non-lesional *IL19* versus EASI severity category (r = −0.3312; *p* = 0.0641). (**C**) Lesional *IL37* versus sleep-disturbance NRS score (r = 0.4086; *p* = 0.0203). (**D**) Lesional versus clinically non-lesional *IL37* (r = 0.4443; *p* = 0.0109). *p* < 0.05 was considered significant and is represented in red.

Exploratory Spearman correlation analyses were conducted separately in the full AD cohort (*n =* 32), and the reported *p*-values were nominal. Lesional *IL19* transcript abundance showed a weak inverse correlation with EASI severity category (*r* = −0.3568; *p* = 0.0450; Figure 4A). A similar inverse correlation was observed for clinically non-lesional *IL19* transcript abundance, although it did not reach statistical significance (*r* = −0.3312; *p* = 0.0641; Figure 4B).

Lesional *IL37* transcript abundance showed a moderate positive correlation with the patient-reported sleep-disturbance NRS score (*r* = 0.4086; *p* = 0.0203; Figure 4C). Lesional and clinically non-lesional *IL37* transcript abundance were also moderately positively correlated with each other (*r* = 0.4443; *p* = 0.0109; Figure 4D).

## 3. Discussion

In this exploratory study, *IL19* and *IL37* showed different expression patterns in superficial epidermal material from patients with AD. *IL37* transcript abundance was higher in lesional skin than in healthy control skin in the overall cohort and both severity strata; increased abundance in lesion-adjacent clinically non-lesional skin was also detected. In contrast, overall *IL19* upregulation was not detected, while lower lesional *IL19* abundance was observed in the severe-to-very severe subgroup. Lesional *IL19* was inversely associated with EASI severity category in the exploratory correlation analysis. However, these observations describe two biologically distinct cytokines within the same clinical and sampling context and do not establish direct interaction or functional pathway activity.

The parallel assessment of both transcripts in the same participants, anatomical regions, and biological material facilitates interpretation of their respective patterns. Comparisons between separate IL-19 and IL-37 studies are complicated by differences in age, disease history, previous treatment, local lesion phenotype, sampling depth, and analytical methods. A common within-study framework reduces some of this variability, while retaining the distinction between IL-19-associated cytokine-responsive keratinocyte signaling and the regulatory functions described for IL-37 [4,5,6,7,8,9,10]. The contribution of this study is therefore a context-specific characterization of these previously investigated transcripts in paired superficial skin compartments, rather than identification of a new cytokine pathway or demonstration of interaction.

### 3.1. Increased IL37 Expression as a Potential Counter-Regulatory Response

The increase in *IL37* transcript abundance in lesional AD skin is compatible with a compensatory response to inflammation, but this interpretation remains a hypothesis. Experimental studies have described complementary intracellular SMAD3-associated and extracellular IL-18Rα/IL-1R8-dependent regulatory mechanisms for IL-37 [9,10]. Hou et al. also linked IL-37b to AMPK–mTOR-associated changes in autophagy in cellular and murine models of allergic inflammation [18]. These findings provide a biological rationale for considering endogenous regulation alongside inflammatory activation; they do not show that the same signaling events occurred in the skin sampled here.

The present study measured mRNA rather than IL-37 protein, secretion, receptor availability, or downstream signaling. Increased transcript abundance may therefore coexist with insufficient translation, altered processing, or ineffective functional regulation. Hou et al. reported reduced circulating IL-37 and reduced IL-18Rα expression in AD [13]. These observations support distinguishing transcriptional induction from effective anti-inflammatory activity, without establishing that a particular receptor or signaling defect explains our result. Paired transcript, protein, and functional measurements would be required to evaluate that possibility.

The earlier skin-based evidence also differs from our observation. Zhou et al. reported reduced *IL37* transcripts and IL-37 protein in AD biopsies and linked IL-37 to differentiated upper-epidermal keratinocytes and barrier-associated proteins [11]. Their direction of change should not be grouped with reports of increased IL-37. Equally, a biopsy-based measurement and the relative transcript signal recovered from superficial tape strip material are not interchangeable outcomes.

Full-thickness biopsies include epidermal and dermal structures and infiltrating cells, whereas tape stripping preferentially recovers stratum corneum-derived and superficial epidermal material. Fritz et al. demonstrated overlapping but non-equivalent transcriptomic profiles in paired tape strips and biopsies and found that several cytokine transcripts, including *IL37*, were better represented in biopsy material [17]. This distinction is relevant when comparing our findings with biopsy studies, but does not itself explain disagreement between studies that both used tape stripping.

Analytical workflows also differ across studies. The earlier pediatric tape strip study used preamplification, TaqMan assays, and normalization to *RPLP0* [14], whereas the present study used SYBR Green RT-qPCR without target-specific preamplification and normalization to *GAPDH* and *ACTB*. These differences are relevant to cross-study comparability but were not evaluated as causes of the discrepant findings. Targeted RT-qPCR, broad transcriptomic profiling, immunohistochemistry, and serum protein assays should therefore be compared with attention to both the analytical method and the biological compartment.

Clinical studies further illustrate that the IL-37 literature does not show a uniform direction of change. Fujita et al. reported increased IL-37 in AD [12], whereas Hou et al. reported reduced circulating IL-37 [13]. Together with the skin findings of Zhou et al. [11], these reports underline the distinction between circulating protein, cutaneous protein, and superficial epidermal mRNA. Our study adds a further transcript-level observation in a different clinical cohort rather than resolving the causes of this variation. Direct mechanistic studies assessing both cytokines together with their downstream pathways in the same biological material will be required to determine whether any functional relationship between IL-19 and IL-37 exists in AD.

### 3.2. IL37 Regulation Beyond Clinically Visible Lesions

The increase in *IL37* transcript abundance in lesion-adjacent clinically non-lesional skin indicates that the observed difference from healthy skin was not confined to visibly eczematous sites. In the overall cohort, *IL37* abundance was higher in both AD compartments than in healthy skin, no significant paired lesional–non-lesional difference was detected, and values in the two AD compartments were moderately positively correlated. Earlier studies have identified immune and barrier abnormalities beyond visible AD lesions [3,14,15]. The present findings are compatible with that broader molecular context, but absence of a significant paired difference is not evidence that the two compartments are biologically equivalent.

The non-lesional samples were collected within 10 cm of an active lesion and should therefore be interpreted as clinically non-lesional, lesion-adjacent skin rather than distant or biologically unaffected skin. Local influence from the neighboring lesion, a wider disease-associated field, and shared systemic or treatment-related factors are possible contributors. The present sampling design cannot distinguish among these explanations.

The difference between healthy and clinically non-lesional skin was observed in the full cohort but was not retained in the age- or severity-stratified analyses. Lesional *IL37* elevation was observed across the overall and stratified comparisons. The lesion-adjacent finding remains of interest, but needs evaluation in studies sampling lesional, adjacent non-lesional, distant non-lesional, and healthy skin within a clearly defined design.

### 3.3. Absence of Uniform IL19 Upregulation

The *IL19* findings provide a contrasting view of superficial epidermal cytokine regulation. Previous studies have demonstrated increased *IL19* mRNA and IL-19 protein in lesional AD biopsy material. Elevated circulating IL-19 has also been associated with EASI, TARC/CCL17, eosinophilia, and clinical improvement during treatment [7,8]. In young children with very early-onset AD, tape strip profiling revealed increased *IL19* expression in lesional and non-lesional skin [14]. These observations have supported a model in which IL-19 reflects cytokine-induced keratinocyte activation and may be associated with both IL-17-related and type 2 inflammatory responses.

Overall *IL19* upregulation was not detected in the present cohort. No significant overall difference was found among healthy, lesional, and lesion-adjacent skin, or within the moderate subgroup and either age stratum. Lower lesional expression was observed in severe-to-very severe AD relative to healthy controls, and lesional *IL19* showed a weak inverse association with EASI severity category. These results do not invalidate earlier observations or establish that IL-19 is unimportant in AD; they identify a different superficial expression pattern in the population studied here.

Several differences warrant consideration without assigning a causal explanation. Acute and chronic AD can differ quantitatively in inflammatory and epidermal transcriptional programs [19,20]. However, the age and morphological stage of the sampled lesions were not systematically documented in this study. The *IL19* result therefore cannot be attributed to chronicity, remodeling, or a particular stage of the local lesion.

The earlier pediatric tape strip cohort comprised children younger than five years with disease duration below six months and showed increased *IL19* together with decreased *IL37* [14]. Thus, the discrepancy from our study concerns both transcripts. Our participants were older children, adolescents, and adults, and do not reproduce that infant and preschool population. Age-associated differences in AD immune and barrier profiles have been demonstrated across childhood and adulthood [21,22], but neither chronological age nor reported age at disease onset can, by itself, explain the differences between these cohorts.

Treatment context also differs between studies. The earlier pediatric study excluded topical anti-inflammatory treatment during the preceding week [14], whereas the present protocol included a 14-day interval. Topical corticosteroid treatment has been shown to modify inflammatory and epidermal gene-expression profiles [23]. Clinical improvement during systemic treatment likewise need not imply complete molecular normalization [24]. These observations support considering previous therapy, but do not establish the persistence of a specific effect on *IL19* or *IL37* after either study’s treatment-free interval.

Previous systemic cyclosporine A use was identified in 14 participants (43.8%) and represents a potential contributor to the observed variation in transcript abundance. Although the protocol required a minimum four-week interval between discontinuation and sampling, the present study cannot determine whether earlier treatment influenced the measured expression patterns. Previous cyclosporine A exposure should therefore be considered part of the clinical context of these findings, rather than an established explanation for them.

Local lesion phenotype is a separate consideration. Although anatomical sampling was standardized, global EASI describes overall disease burden rather than the age or morphology of an individual sampled lesion. Transcriptomic work has associated local erythematous, indurated, and lichenified phenotypes with different molecular signatures [25]. Such variation may be relevant to measurements reported, but no retrospective acute-versus-chronic or erythematous-versus-lichenified classification was assigned. Overall disease history, disease severity at sampling, and local lesion characteristics should therefore remain distinct in interpreting these findings.

**Figure 5:**
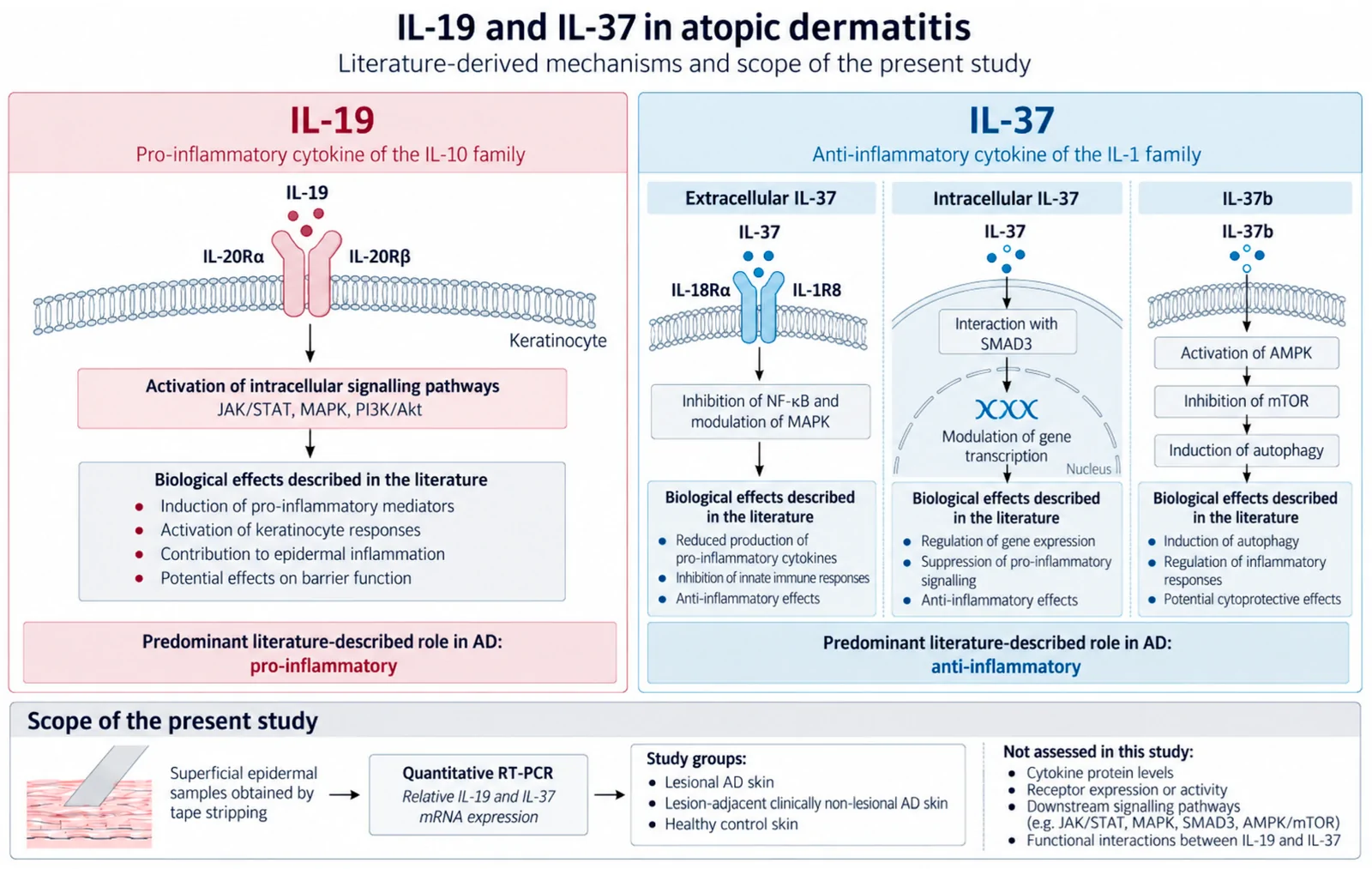
Literature-derived mechanisms of IL-19 and IL-37 in atopic dermatitis and scope of the present study. Selected IL-19- and IL-37-related pathways described in previous studies are presented as parallel, biologically distinct mechanisms and should not be interpreted as evidence of a direct mechanistic interaction between IL-19 and IL-37. These pathways were not experimentally assessed in the present cohort, which evaluated relative superficial epidermal *IL19* and *IL37* mRNA abundance in tape-strip samples.

To provide a visual overview without implying a unified signaling cascade, the literature-derived mechanisms discussed above are summarized in Figure 5 as parallel, biologically distinct pathways; the experimental scope of the present study is shown separately.

### 3.4. Clinical and Age-Related Associations

The positive association between lesional *IL37* transcript abundance and the sleep-disturbance NRS score is an exploratory finding whose biological meaning remains uncertain. A cross-sectional association cannot determine whether inflammatory stress influences both variables, whether regulatory responses are involved, or whether other clinical factors account for the relationship. It does not establish that IL-37 causes sleep disturbance or constitutes a marker of symptom burden.

The wider neuroimmune context is discussed in the review by Łacwik et al., which considers the IL-31/IL-33 axis in relation to type 2 inflammation, epithelial alarmin signaling, and the itch–scratch cycle [26]. A potential link with endogenous regulation is provided by Tsuji et al., who showed that IL-37 contributes to reduced IL-33 expression following AHR activation in human keratinocytes [27]. These publications provide a rationale for investigating regulatory and itch-associated pathways together. However, they do not explain the correlation observed here since IL-31 and IL-33 were not measured, and mediation through this pathway was not evaluated.

Age-stratified analyses showed increased lesional *IL37* relative to the corresponding healthy-control group in both participants younger than 18 years and adults. These findings extend the lesional observation across the age strata examined; they do not establish that its magnitude increases with age or that age modifies the association.

No significant *IL19* differences were detected within either age stratum. This finding does not identify chronological age as the explanation for the discrepancy from early pediatric studies. Larger and prospectively characterized cohorts would be needed to distinguish age-related biology from differences in disease history, local lesions, and treatment exposure.

### 3.5. Strengths and Limitations

The study assessed both transcripts in the same participants, anatomical regions, and superficial epidermal samples. Paired lesional and clinically non-lesional sampling enabled within-patient comparisons, while healthy-control skin provided a reference for disease-associated differences. These features allow the respective expression patterns of *IL19* and *IL37* to be described within a common clinical framework. Tape stripping also offers a practical basis for repeated superficial molecular sampling without a full-thickness biopsy.

The principal limitations are the exploratory, single-center, cross-sectional design and the relatively small cohort, particularly after stratification. Clinical correlations require independent confirmation and should not be treated as validated biomarkers. Disease-onset information was recorded as broad patient-reported life-stage categories rather than exact ages or dates; therefore, exact disease duration could not be calculated or used in the analyses. The recorded onset categories are presented descriptively in Table 1 and Table 2. The duration and morphological stage of individual sampled lesions were not systematically documented; chronological age, onset category, and global EASI do not replace lesion-level characterization. Anatomical standardization did not remove that source of uncertainty. In addition, the clinically non-lesional samples were lesion-adjacent and cannot be assumed to represent distant uninvolved skin.

Previous cyclosporine A exposure in a subset of participants is a potential confounder. The cross-sectional design did not allow its contribution to the observed *IL19* and *IL37* expression patterns to be determined.

RT-qPCR measured relative transcript abundance without isoform-specific resolution. Cytokine protein, secretion, receptor expression, post-translational processing, and functional activity were not measured. The biological mechanisms discussed above are literature-based interpretations rather than results of pathway testing in this cohort. Longitudinal studies integrating transcript, protein, local lesion, and treatment information will be needed to evaluate their relevance.

## 4. Materials and Methods

### 4.1. Study Design and Participants

This exploratory, single-center, cross-sectional observational study included participants recruited between March 2021 and November 2025 from the outpatient clinics of the Department of Dermatology, Venereology and Allergology, Medical University of Gdańsk, Gdańsk, Poland. The final analytical cohort comprised 32 patients with atopic dermatitis (AD) and 35 healthy controls. The groups were not individually matched. Paired tape strip samples from lesional and clinically non-lesional skin were available for all patients with AD. Summary characteristics of the study population are presented in Table 1.

AD was diagnosed by a dermatologist according to the Hanifin and Rajka criteria [28]. Healthy controls had no current or previous diagnosis of AD, no history of other allergic diseases, autoimmune diseases, or malignancies, and no active inflammatory skin disease at the sampling site. All participants were of Central European descent.

Participants receiving systemic immunosuppressive or immunomodulatory treatment or phototherapy at the time of sampling, as well as those with a clinically evident skin infection, were excluded. Previous systemic treatment did not constitute an exclusion criterion and was recorded. Previous systemic cyclosporine A use was identified in 14 of the 32 patients with AD (43.8%), based on available medical records and patient-reported treatment histories. The study protocol required cyclosporine discontinuation at least four weeks before clinical assessment and tape strip sampling. Disease severity was assessed using EASI at the sampling visit and therefore does not represent pretreatment severity. Systemic corticosteroids had been discontinued for at least 4 weeks before clinical assessment and sampling, and topical anti-inflammatory treatment had been withheld for at least 14 days.

The study was conducted in accordance with the Declaration of Helsinki and was approved by the Independent Bioethics Committee for Scientific Research at the Medical University of Gdańsk (approval no. NKBBN/209/2021; approval date: 5 March 2021). Recruitment, clinical assessments, and sample collection began in March 2021, after this approval had been obtained. Written informed consent was obtained from all adult participants. For participants younger than 18 years, written informed consent was obtained from a parent or legal guardian, together with age-appropriate assent where applicable.

### 4.2. Clinical and Laboratory Assessment

Demographic and disease-related data collected at the sampling visit included age, sex, reported age-at-onset category, previous systemic treatment, and the presence of allergic/atopic and non-atopic comorbidities. Allergic/atopic comorbidities included bronchial asthma, allergic rhinitis, allergic conjunctivitis, food allergy, contact allergy, inhalant allergy, and oral allergy syndrome, where recorded; other chronic medical conditions were recorded separately as non-atopic comorbidities.

Patient-reported age at AD onset was available as broad categories (infancy, early childhood, or adolescence), rather than exact ages or dates. Because exact onset ages were unavailable, patient-level disease duration in years was not reconstructed. The recorded onset categories are summarized in Table 1 and presented for individual participants in Table 2; they were treated as descriptive clinical characteristics rather than exact duration measurements.

Pruritus intensity and sleep disturbance were each assessed using an 11-point numerical rating scale (NRS) ranging from 0 (no symptoms) to 10 (greatest symptom severity).

AD severity was assessed on the day of skin sampling using the Eczema Area and Severity Index (EASI) and recorded according to established severity strata: clear, 0; almost clear, 0.1–1.0; mild, 1.1–7.0; moderate, 7.1–21.0; severe, 21.1–50.0; and very severe, 50.1–72.0 [29]. For severity-stratified group comparisons, the severe and very severe strata were combined into a single severe-to-very severe group. The analytical cohort included 16 patients with moderate AD and 16 patients with severe-to-very severe AD. For the exploratory correlations shown in Figure 4, the observed EASI severity categories (moderate, severe, and very severe) were treated as an ordinal variable.

The available laboratory information comprised categorical eosinophilia status rather than numerical eosinophil counts. Eosinophilia was defined as an absolute peripheral blood eosinophil count greater than 0.45 × 10^9^/L. The recorded status is presented in Table 2 alongside the individual clinical characteristics. For exploratory age-stratified analyses, participants were categorized as younger than 18 years or 18 years and older.

### 4.3. Transepidermal Water Loss Measurement

Transepidermal water loss (TEWL) was measured using a VapoMeter (Delfin Technologies Ltd., Kuopio, Finland) under ambient room conditions. Measurements were obtained at the lesional and clinically non-lesional antecubital sampling sites in patients with AD and at the anatomically corresponding antecubital site in healthy controls. Three readings were obtained at each measurement site, and their arithmetic mean was used as the reported TEWL value expressed as g/m^2^/h.

### 4.4. Tape-Stripping Procedure

Tape stripping was used to collect stratum corneum-derived material for superficial epidermal transcript analysis through sequential application and removal of adhesive sampling discs. Large D-Squame^®^ discs (D102; CuDerm Corporation, Dallas, TX, USA) were used. In patients with AD, samples were obtained from an active eczematous lesion in the antecubital fossa and nearby clinically non-lesional, lesion-adjacent skin within the same anatomical region. Healthy control samples were obtained from clinically normal skin at the anatomically corresponding antecubital site. Throughout the manuscript, this AD compartment is referred to as clinically non-lesional skin.

Twenty consecutive tape strips were collected from each sampling site. All discs were applied sequentially to the same predefined skin area. Each disc was pressed against the skin for 5 s using a D-Squame® Pressure Instrument D500 (CuDerm Corporation, Dallas, TX, USA), which applied a standardized pressure of 225 g/cm^2^, and was then removed using a rapid, uniform motion [30].

Immediately after removal, the discs were transferred into sterile collection tubes. Samples from each site were maintained as separate, site-specific sets, placed immediately on dry ice, and subsequently stored at −80 °C until RNA isolation. Lesional, clinically non-lesional, and healthy control samples were labeled and handled separately. Of the 20 strips collected at each site, ten were pooled for RNA extraction and processed as one biological sample, as described in Section 4.5. Remaining tape strips were stored as backup material in case additional extraction was needed.

### 4.5. RNA Isolation and Quality Control

Epidermal material on the ten pooled tape strips was lysed directly using RLT buffer (QIAGEN, Hilden, Germany) supplemented with β-mercaptoethanol (Sigma-Aldrich, St. Louis, MO, USA). Total RNA was extracted using the RNeasy Mini Kit (QIAGEN, Hilden, Germany) according to the manufacturer’s protocol. RNA concentration and purity were assessed spectrophotometrically using a DS-11+ spectrophotometer (DeNovix, Wilmington, DE, USA).

### 4.6. Reverse Transcription and RT-qPCR

Total RNA was reverse-transcribed into complementary DNA (cDNA) using the QuantiTect Reverse Transcription Kit (QIAGEN, Hilden, Germany), including the kit-integrated genomic DNA elimination step, according to the manufacturer’s protocol. No target-specific preamplification was performed.

Relative *IL19* and *IL37* mRNA abundance was quantified from the resulting cDNA by reverse-transcription quantitative PCR (RT-qPCR) using the QuantiNova SYBR Green PCR Kit (QIAGEN, Hilden, Germany) on a CFX96 Real-Time PCR Detection System (Bio-Rad Laboratories, Hercules, CA, USA). Each biological sample was analyzed in three technical replicates. Negative controls were included, and amplicon specificity was evaluated by melting-curve analysis.

The suitability of *GAPDH* and *ACTB* as reference genes was evaluated using BestKeeper v1.0 [31]. Target-specific primer sequences are provided in Table 3. Primer specificity was assessed in silico using NCBI Primer-BLAST [32]. The assays measured *IL19* and *IL37* transcript abundance without distinguishing individual transcript isoforms.

### 4.7. Relative Gene-Expression Analysis

Relative transcript abundance was analyzed separately for *IL19* and *IL37* using the comparative Cq method. For each sample and target, ΔCq was calculated as the target-gene Cq minus the mean Cq of *GAPDH* and *ACTB*: ΔCq = Cq_target_ − mean(Cq*_GAPDH_*, Cq*_ACTB_*). For each target, the mean ΔCq of the healthy-control group served as the calibrator: ΔΔCq = ΔCq_sample_ − mean(ΔCq_HC_), where HC denotes healthy controls. Fold change was calculated as 2^−ΔΔCq^, and log_2_FC was calculated as log_2_(fold change), equivalent to −ΔΔCq. Higher log_2_FC values indicated greater transcript abundance relative to the healthy-control reference.

For samples without detectable target-specific amplification, a Cq value of 50 was assigned for calculation of relative expression. Because *IL19* and *IL37* were measured using separate target-specific assays, their raw Cq values and absolute transcript levels were not compared directly.

### 4.8. Study Outcomes

The co-primary outcomes were differences in relative *IL19* and *IL37* transcript abundance among lesional AD skin, clinically non-lesional AD skin, and healthy control skin.

Secondary outcomes included paired intraindividual differences between lesional and clinically non-lesional AD skin and exploratory associations of relative transcript abundance with EASI severity category, pruritus NRS, sleep-disturbance NRS, TEWL, age, sex, reported age-at-onset category, peripheral blood eosinophilia, and allergic/atopic and non-atopic comorbidities. Additional exploratory outcomes included expression patterns according to disease-severity and age strata and the relationship between lesional and clinically non-lesional *IL37* abundance. All subgroup and association analyses were considered exploratory.

### 4.9. Statistical Analysis

Statistical analyses were performed using GraphPad Prism 10 (GraphPad Software, San Diego, CA, USA). Distributional normality was assessed using the Shapiro–Wilk test. For comparisons among healthy control, lesional AD, and clinically non-lesional AD skin, one-way analysis of variance followed by Tukey’s multiple-comparisons test or the Kruskal–Wallis test followed by Dunn’s multiple-comparisons test was used, as appropriate. Paired lesional-versus-non-lesional comparisons were performed using the paired *t*-test or Wilcoxon matched-pairs signed-rank test, according to the distribution of the paired data. The *p*-values reported for multiple-group comparisons were adjusted using the corresponding post hoc procedure.

Associations between relative transcript abundance and clinical variables were evaluated using separate Spearman rank-correlation analyses in the full AD cohort (n = 32). These analyses were exploratory, and nominal *p*-values are reported. All tests were two-sided, and *p* < 0.05 was considered statistically significant.

### 4.10. Use of Generative Artificial Intelligence

During the preparation of this manuscript, ChatGPT Pro, version 6 (OpenAI, San Francisco, CA, USA) was used for language editing and to assist in the preparation of the literature-derived conceptual schematic (Figure 5). The tool was not used for study design, data collection, statistical analysis, or interpretation of the study results. All text and graphical output were reviewed and edited by the authors.

## 5. Conclusions

In this cohort, superficial epidermal *IL19* and *IL37* showed distinct expression patterns. *IL37* transcript abundance was increased in lesional AD skin in the overall cohort and across the examined severity and age strata; an increase in lesion-adjacent clinically non-lesional skin was also observed in the overall analysis. Overall *IL19* upregulation was not detected, while lower lesional *IL19* abundance was observed in severe-to-very severe AD. The exploratory clinical associations provide additional questions for investigation rather than validated markers of disease activity or symptoms.

These findings extend the characterization of two biologically distinct cytokines to paired superficial skin compartments in older children and adults. Increased *IL37* transcription is compatible with a compensatory response, but does not establish effective anti-inflammatory signaling or direct antagonism with IL-19. The observations should be interpreted within the clinical, sampling, and previous-treatment context of this cohort. Independent and longitudinal studies integrating transcript isoforms, protein abundance, receptor signaling, local lesion characterization, and treatment history are needed to establish their biological and clinical significance.

## Figures and Tables

**Table 1:** Summary characteristics of the study population.

Variable	Category	AD Full Cohort (N = 32)	AD Moderate EASI (N = 16)	AD Severe/Very Severe EASI (N = 16)	Healthy Control (N = 35)
Sex	Male ^1^	20 (62.5%)	10 (62.5%)	10 (62.5%)	18 (51.4%)
	Female ^1^	12 (37.5%)	6 (37.5%)	6 (37.5%)	17 (48.6%)
Age	Full cohort ^2^	29.2 (8–64; 15.7)	32.6 (16–64; 14.4)	25.9 (8–54; 16.2)	21.4 (6–43; 8.8)
	<18 years	N = 13	N = 4	N = 9	N = 13
	≥18 years	N = 19	N = 12	N = 7	N = 22
Reported AD onset ^3^	Infancy	10 (31.3%)	1 (6.3%)	9 (56.3%)	—
	Early childhood	20 (62.5%)	13 (81.3%)	7 (43.8%)	—
	Adolescence	2 (6.3%)	2 (12.5%)	0 (0.0%)	—

^1^ n (%); ^2^ mean (minimum–maximum; SD). ^3^ n (%); onset category denotes the patient-reported life stage at AD onset (infancy, early childhood, or adolescence). Exact age or date of onset was not recorded; therefore, exact patient-level disease duration was not reconstructed.

**Table 2:** Detailed characteristics of patients with atopic dermatitis.

ID	Age	Sex	EASI	Pruritus ^1^	Sleep Disturbance ^1^	TEWL ^2^	Onset	Eosinophilia	Allergic/Atopic Comorbidities ^3^	Non-Atopic Comorbidities
41	23	male	moderate	5	7	41.8/31.2	early childhood	+	A	?
42	15	female	severe	10	7	64.3/35.3	early childhood	−	AR; AC; A	?
43	64	male	moderate	7	7	56.7/34.2	adolescence	−	-	Type 2 diabetes; hypertension; obstructive sleep apnea; purulent pericarditis; diabetic neuropathy; cataract; diabetic maculopathy
44	16	female	moderate	7	9	49.7/24.3	early childhood	+	-	?
45	53	male	moderate	7	6	37.8/24.1	adolescence	−	AR; A	?
46	27	male	moderate	5	5	42.3/21.5	early childhood	−	-	-
47	48	male	severe	8	6	56.3/34.6	early childhood	−	AR; A	obstructive sleep apnea; Sjögren syndrome; AVNRT; autoimmune vasculitis; obesity; post-atrioventricular (dal-free) pathway ablation; post-MI/NSTEMI;
48	23	female	moderate	7	7	52.4/25.6	early childhood	−	-	conductive hearing loss; tympanoplasty
49	34	female	moderate	4	7	26.3/17.8	early childhood	−	A	?
50	16	male	moderate	6	7	32.8/28.2	early childhood	−	-	?
51	54	female	moderate	6	9	51.3/26.7	early childhood	+	-	-
52	16	female	moderate	5	8	36.2/21.3	early childhood	−	A	?
53	44	male	moderate	7	8	38.5/27.1	early childhood	−	-	hypertension; deep vein thrombosis
56	11	female	severe	5	10	47.2/26.1	early childhood	+	AR; A	reactive hypoglycemia
57	14	male	severe	6	6	42.3/24.7	infancy	−	A	-
58	16	female	moderate	6	4	47.8/23.1	early childhood	+	A	-
59	35	male	moderate	3	3	46.8/27.5	early childhood	+	-	pneumothorax
60	13	female	severe	5	0	63.2/37.8	infancy	+	AR	anemia
61	34	male	severe	8	8	49.5/24.5	infancy	?	-	erysipelas
62	22	male	very severe	7	7	62.6/26.7	infancy	+	A	-
63	34	male	moderate	7	7	48.4/28.7	early childhood	−	AR; A	-
64	14	male	severe	6	1	42.1/25.4	infancy	−	AR; A	-
65	8	female	severe	7	3	61.5/28.3	early childhood	+	AR; CA; IA; AC; OAS	depression
66	14	male	severe	6	6	42.3/24.7	infancy	−	A	-
67	16	male	severe	8	8	45.3/26.3	infancy	+	AR; A	-
68	48	male	severe	4	1	35.7/27.3	early childhood	+	AR	-
70	32	male	moderate	2	1	31.5/17.5	early childhood	+	A	depression
72	54	female	severe	7	7	56.2/33.5	infancy	−	A	hypertension; fatty liver disease; degenerative spine disease
73	34	male	severe	8	8	63.2/37.8	infancy	−	AR; A	hypertension
74	54	male	very severe	7	6	55.7/25.8	early childhood	−	-	hypertension; fatty liver disease; degenerative spine disease
76	34	male	moderate	3	3	39.8/25.8	infancy	+	AR	-
77	15	female	severe	10	7	64.3/35.3	early childhood	−	AR; AC; A	?

^1^ 0–10 rating scale (0 indicates no symptoms and 10 indicates the greatest symptom severity); ^2^ transepidermal water loss in g/m^2^/h by sampling site (lesional/non-lesional); ^3^ A—asthma, AR—allergic rhinitis, AC—allergic conjunctivitis, CA—contact allergy, IA—inhalant allergy, OAS—oral allergy syndrome. Onset denotes the reported life stage at AD onset; + and − indicate presence and absence, respectively; ? indicates unavailable information.

**Table 3:** Primer sequences used in the study.

Target	Forward Primer, 5′–3′	Reverse Primer, 5′–3′
*IL19*	CGTTCTACGTGGACAGGGTGTT	CAGTGACACTGCCTCTGTTCCT
*IL37*	TCAGCCTCTGCGGAGAAAGGAA	GCGTGCTGATTCCTTTTGGGCA
*GAPDH*	GTCTCCTCTGACTTCAACAGCG	ACCACCCTGTTGCTGTAGCCAA
*ACTB*	CACCATTGGCAATGAGCGGTTC	AGGTCTTTGCGGATGTCCACGT

## Data Availability

The data supporting the findings of this study are available from the corresponding author upon reasonable request. The data are not publicly available due to participant privacy considerations.
